# Radiation skyshine from a 6 MeV medical accelerator

**DOI:** 10.1120/jacmp.v11i3.3032

**Published:** 2010-05-06

**Authors:** Michael S. Gossman, Patton H. McGinley, Mary B. Rising, A. Jussi Pahikkala

**Affiliations:** ^1^ Tri‐State Regional Cancer Center Medical Physics Section 706 23rd Street Ashland KY 41101 USA; ^2^ 685 Tahoe Circle Stone Mountain GA 30083 USA; ^3^ Department of Mathematics University of Louisville Louisville KY 40292 USA; ^4^ FI‐21310 Vahto Finland

**Keywords:** scattering, shielding, skyshine, solid angle

## Abstract

This study assesses the dose level from skyshine produced by a 6 MeV medical accelerator. The analysis of data collected on skyshine yields professional guidance for future investigators as they attempt to quantify and qualify radiation protection concerns in shielding therapy vaults. Survey measurements using various field sizes and at varying distances from a primary barrier have enabled us to identify unique skyshine behavior in comparison to other energies already seen in literature. In order to correctly quantify such measurements outside a shielded barrier, one must take into consideration the fact that a skyshine maximum may not be observed at the same distance for all field sizes. A physical attribute of the skyshine scatter component was shown to increase to a maximum value at 4.6 m from the barrier for the largest field size used. We recommend that the largest field sizes be used in the field for the determination of skyshine effect and that the peak value be further analyzed specifically when considering shielding designs.

PACS numbers: 87.52.‐g, 87.52.Df, 87.52.Tr, 87.53.‐j, 87.53.Bn, 87.53.Dq, 87.66.‐a, 89., 89.60.+x

## I. INTRODUCTION

Skyshine radiation emanating from medical accelerator facilities is a phenomenon not well understood. A comprehensive analysis of skyshine has not been reported in the applicable literature for 6 MV X‐rays by the NCRP in Reports 49, 51, 79 or 147 or the Institute of Physics and Engineering in Medicine in Report 75.^(^ ^1^ ^,^ ^2^ ^,^ ^3^ ^,^ ^4^ ^,^ ^5^ ^)^ Where literature is available for skyshine, it exists for higher energy X‐ray beams only.^(^ ^6^ ^,^ ^7^ ^,^ ^8^ ^)^ It is defined by the National Council on Radiation Protection and Measurement as radiation scattered back to Earth by the atmosphere above a radiation‐producing facility.^(^ ^6^ ^)^ The purpose here is to determine the appropriate technique for separating skyshine measurements from leakage and scatter radiation transmitted through the wall adjacent to the area where the measurements were made. Skyshine measurements are obtained in this research from a clinically operating facility.

## II. MATERIALS AND METHODS

Data was obtained at a facility housing a Varian Medical Systems, Inc. (Palo Alto, CA) Model 6EX particle accelerator. Radiation measurements were taken in the adjacent parking lot, immediately lateral to the position of the isocenter. This vault wall constitutes a primary barrier for the facility. The total distance from the isocenter of the machine to the barrier is 2.44 m. The barrier includes only 0.51 m of concrete; an amount needed for an operational workload at about a third of typical capacity. Thus, the total distance from the isocenter to the outside of the barrier is 2.95 m. The ceiling of the vault is also shielded with 0.51 m of concrete. This original shielding design provided for a distance of 2.3 m from the machine isocenter to the exterior surface of the roof. This arrangement is similar to that published in NCRP Report 151 or originally published by McGinley.^(^ ^6^ ^,^ ^7^ ^,^ ^8^ ^)^

A pressurized ionization chamber (Fluke Biomedical, Cleveland, OH, Model 451P) was chosen for all measurements. The device was identified as being suitable due to its fast response time to radiation with capabilities that include auto‐ranging, auto‐zeroing, high X‐ray sensitivity and resolution of exposure rate down to 10.32 nC kg‐1 hr‐1 (40 μR h‐1).

The linear accelerator gantry was positioned so the X‐ray beam was directly toward the ceiling of the vault. For International Electrotechnical Commission (IEC) 61217 geometry scaling, the gantry angle was 180°, with collimator angle and couch angle each set at 0°.^(^ ^9^ ^)^ Annual machine calibration was completed prior to this work, resulting in $1.00\;{\text{Gy MU}}^{-1}$ delivery 100 cm from the source, at the depth of maximum dose clinically in a $10\times 10\;{\text{cm}}^{2}$ beam. At the machine isocenter, the accelerator was programmed at a fixed dose rate of $6.67\text{cGy}\;s^{-1}(400\;\text{MU}\;\min^{-1})$. The Varian Model Millennium 120‐leaf multileaf collimator (MLC) was fully retracted in standby mode during each survey. The square field size was set to $40\times 40\;{\text{cm}}^{2}$ initially.

Measurements were made in a vacant parking lot adjacent to the therapy facility. The detector remained in the transverse central‐axis plane (gantry plane) of the linear accelerator at all times. Each measurement was taken at a fixed height of 1.8 m above the ground. The detector was pointed towards the roof at a point observed to be 2 m above the edge of the building, so as to detect air scattering radiation emanating from the roof of the building. For all measurements in this study, no phantom was required to be placed at isocenter.

In order to consider potential relationships which might exist between our readings and the distance from the lateral barrier, measurements were taken in 1.52 m (5 ft) increments. Between the initial distance of 1.52 m and the final distance of 15.2 m, a total of 10 measurements were taken. As found in IPEM Report 75, the mean energy of skyshine radiation has been reported to be between 120–250 keV.^(^ ^5^ ^)^ Based on this energy range, the ratio of absorbed dose to exposure is $3.76\mu \text{Sv}\;{\text{nC}}^{-1}\;\text{kg}(0.97\;\text{rem}\;R^{-1})$.^(^ ^10^ ^)^ Since the ratio is almost one, it was assumed that the exposure rate was identical to the dose rate. Likewise, in order to consider potential relationships which might exist between our readings and field size in use, this process was repeated for a variety of field sizes at each distance. The open field beam sizes considered were $5\times 5\;{\text{cm}}^{2}$, $10\times 10^{2}$, $20\times 20\;{\text{cm}}^{2}$, $30\times 30\;{\text{cm}}^{2}$ and $40\times 40\;{\text{cm}}^{2}$.

Leakage radiation can be measured separately as a function of distance from isocenter since only the leakage component contributes at $0\times 0\;{\text{cm}}^{2}$ field size. Skyshine is calculated as the difference between these two measurements, the one with a non‐zero field size and the other with a $0\times 0\;{\text{cm}}^{2}$ field size. Leakage and scatter radiations penetrating the lateral barrier and contributing to the total are very different in radiation quality. Leakage radiation can be separately measured as a function of distance from isocenter. If the field size is reduced, both the skyshine and scatter components are reduced. Only leakage contributes at 0 cm field size, assuming the scatter component is negligible. Therefore, the radiation exiting the roof is reduced. Each measurement taken includes the skyshine component as well as the combined scatter and leakage. Skyshine is calculated as the difference between these two measurements.

In order to identify the level of these two components, the X‐ray beam size was reduced to the smallest possible, $0.4\;\times \;0.4\;{\text{cm}}^{2}$. The narrow beam remaining was blocked by placing a 7 cm thick by $5\times 5\;{\text{cm}}^{2}$ cross‐sectional cerrobend block in the tray slot. Again, measurements were performed at each stated distance with the same detector positioning. Skyshine at each distance was then calculated as the difference between the measurement for the field size set and the measurement for the blocked $0.4\;\times \;0.4\;{\text{cm}}^{2}$ beam.

Measurements were then converted to the appropriate units and tabulated for analysis. The accuracy of skyshine calculations, based on the methodology given below, was compared to measured results. The shape of the graphs plotted revealed information regarding inaccuracies as well as distinct behavior for a 6 MV X‐ray beam in comparison to 18 MV X‐ray beam results already found in published research.

Here, steps are followed as published which lead directly to the mathematical result for skyshine.^(^ ^6^ ^,^ ^8^ ^)^ First, the amount of X‐ray radiation likely to penetrate the shielded vault ceiling defines the total transmission factor ($B_{\text{xs}}$). It can be calculated from

(1) $$B_{xs}=10^{-\left\{1+\left[\frac{\left(t-TVL_{1}\right)}{TVL_{e}}\right]\right\}}$$

as in NCRP‐151.^(^ ^6^ ^)^ Variables ${\text{TVL}}_{1}$ and equilibrium ${\text{TVL}}_{e}$ are the first and second tenth‐value layers of the desired material. The total transmission factor can then be calculated from Eq. 1. It was identified that for an endpoint energy of 6 MV, the primary barrier tenth‐value layers were 37 cm for ${\text{TVL}}_{1}$ and 33 cm for ${\text{TVL}}_{e}$ for concrete (NCRP 2005). Restated, the original vault ceiling was shielded by 0.51 m of concrete. Using this information, the resulting barrier transmission ($B_{\text{xs}}$) was determined to be 0.038.

The dose‐equivalent rate $\dot{H}(\text{nSv}\;h^{-1})$ to be measured for skyshine is directly dependent on the transmission through the barrier.^(^ ^8^ ^)^ Equation 2 shows the calculation required to be in the form

(2) $$\dot{H}=\frac{2.5\times 10^{7}\left(B_{xs}{\dot{D}}_{o}\Omega^{1.3}\right)}{{\left(d_{i}d_{s}\right)}^{2}}$$

where ${\dot{D}}_{{}^{\circ}}(\text{Gy}/h)$ represents the X‐ray absorbed dose output at a distance of 1 m from the target, vertical distance $d_{i}$ (m) from the target to a point 2 m above the roof, and lateral distance $d_{s}$ (m) from the isocenter to a point outside the barrier where measurements are taken. The variable Ω (steradians) defines the solid angle formed by the radiation beam.

During this experiment, the X‐ray absorbed dose rate was unchanged. A constant value for was held for all measurements at $6.67\;\text{cG}\;s^{-1}$. The orientation of the particle accelerator and vault room shielding did not change either, leaving $d_{i}$ also constant at 5.3 m. The value of the variable $d_{s}$ changed for each incremental position of measurement in the parking lot. The solid angle changed as well for each field size used.

## III. RESULTS

**Figure 1:**
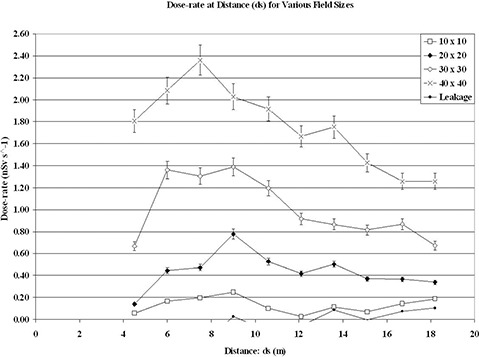
Skyshine measured dose rates at 6 MV plotted as a function of distance for various field sizes.

Measurements obtained for the skyshine component result are presented in Fig. 1. It is immediately observable from the form of the first plot that skyshine increases rapidly with distance outside the barrier ($d_{s}=2.95\;m$) to a peak, very distinct at large field sizes, and then falls off gradually. It is conversely observed that this peak becomes less distinct for smaller field sizes. For a $40\times 40\;{\text{cm}}^{2}$ field size, the peak occurs at a distance of around $d_{s}=7.2\;m$. A second peak appears for the $40\times 40\;{\text{cm}}^{2}$ field size results. The origin of the two peaks is best understood by assuming that skyshine radiation first increases in value due to partial penetration of the roof edge. After some finite distance, skyshine radiation is dominated by the effect of $1/r^{2}$ intensity fall‐off as the distance from isocenter increases. For the purposes of radiation safety and the “as low as reasonably achievable” (ALARA) principal, one should make use of the dose rate involving the highest peak. For a $10\times 10\;{\text{cm}}^{2}$ field size, the greatest peak occurs at a distance of around $d_{s}=8.9\;m$, which is about 5.9 m from the lateral barrier. Another peak is also noted. However, in this geometry we are less interested in this graphical location as it does not present the peak for maximum dose measurement from skyshine in the public parking area. The figure indicates the expected increase with field size.

**Figure 2:**
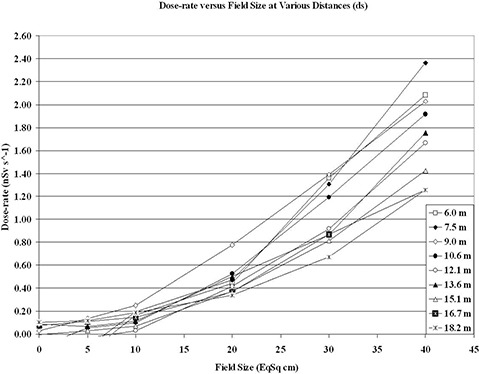
Skyshine measured dose rates at 6 MV plotted as a function of field size for various distances.

Measured dose rates at 6 MV are shown in Fig. 2, plotted as a function of field size from the isocenter for various distances. Again, the skyshine dose rate is represented by the difference between the measured dose rate at the given field size and the reading resulting from a completely blocked field (i.e. $0\times 0\;{\text{cm}}^{2}$). No peak pattern was observed for this plot. As in Fig. 1, an increase in skyshine with respect to all field sizes was noted. Skyshine at further distances was found to be less than measured results closer to the primary lateral barrier. For smaller field sizes, few differences were noted between skyshine readings at any distance measured. Larger differences occurred at larger field sizes, with the largest difference occurring for a $40\times 40\;{\text{cm}}^{2}$ field size between distances 7.5 m and 18.2 m from the isocenter. The resulting dose rate difference for skyshine was then 1.11 nSv/s over this distance range. It is deduced that the atmospheric scattering relationship is a function of field size to some second order magnitude of a polynomial.

## IV. DISCUSSION

We attribute no error to the positioning of the ionization instrument. Technical data sheets on the Fluke Biomedical Model 451P‐RYR device indicate an angular correction of 1.000 through 0° to 60° of orientation for higher energy X‐rays.^(^ ^11^ ^)^ According to the most recent calibration of the device, exposure‐rate calibration ranges are accurate to within 2.2%.^(^ ^12^ ^)^ The uncertainty of the source used for its calibration was assigned 3.6%. Within the lower ranges of dose rate, from $0-5\;\mu \text{Sv}\;{\text{hr}}^{-1}(0-1.4\;\text{nSv}\;s^{-1})$ and $5-50\;\mu \text{Sv}\;{\text{hr}}^{-1}(1.4-14\;\text{nSv}\;s^{-1})$, the cumulative error assigned is ±5.8%. This is indicated in Fig. 1.

As similarly seen with a study involving an 18 MV particle accelerator, there is meager agreement between the calculated and measured values for skyshine.^(^ ^7^ ^,^ ^8^ ^)^ In our study, Eq. 2 was also found to underestimate the dose rate from skyshine at nearly all distances. For field sizes of $20\times 20\;{\text{cm}}^{2}$, $30\times 30\;{\text{cm}}^{2}$ and $40\times 40\;{\text{cm}}^{2}$, the ratio resulted in a dose rate three times different in magnitude. At $10\times 10\;{\text{cm}}^{2}$, the magnitude changes to within a factor of ten, with the exception of the furthest distance studied. For a field size of $5\times 5\;{\text{cm}}^{2}$ the magnitude of difference oscillates between underestimation and overestimation dramatically, with less discrepancy at shorter distance, up to a factor of over forty at the furthest distance considered. Previously, for an 18 MV medical accelerator, the peak effect on the skyshine dose rate was also seen, although it occurred at 13.6 m from the isocenter.^(^ ^7^ ^)^ In this study, we have shown that the peak occurs at a different position relative to the barrier for accelerator X‐ray energies of 6 MV. We have also shown that the peak position is a function of the field size employed in this exercise. This behavior appears to be caused by an increase in the scattering cross‐sectional variable when the scattering angle subtended is decreased.

As recommended by NCRP Report No. 151, Eq. 2 should be used with caution.^(^ ^6^ ^)^ It is understood that an order of magnitude may be the best result from such estimates. Research is needed to understand the phenomenon further, although the form of skyshine with respect to field size, distance and two common high‐energy modalities are now possible.

## V. CONCLUSIONS

Roofs are typically areas with restricted or controlled access. These locations are not the only thing to study when conducting shielding experiments for medical accelerator facilities. Radiation may be found at ground level, yet originate in the air above the vault.^(^ ^13^ ^,^ ^14^ ^)^ In order to correctly quantify such measurements outside a shielded barrier, one must take into consideration the fact that a skyshine maximum may not be observed at the same distance for all field sizes. For this reason, it should be an additional measurement to acquire along with the usual 0.3 m measurement outside barriers.^(^ ^6^ ^)^ Data should be compiled from field measurements to determine this atmospheric scattering component.

Survey measurements using various field sizes and at varying distances from a primary barrier have enabled us to identify unique skyshine behavior in comparison to other energies already seen in literature. Larger dose rates are achieved by using large field sizes. A physical attribute of the skyshine scatter component was shown to increase to a maximum value at 4.2 m from the barrier for the largest field size used. Here, we have shown that the equation for calculating skyshine results in better agreement with measurements at larger field sizes for a variety of distances as well. We recommend that the largest field sizes be used in the field for the determination of skyshine effect and that the peak value be further analyzed specifically when considering shielding designs.
